# Relationships between asbestos exposure and pleural plaques: dose and time effects using fractional polynomials

**DOI:** 10.1136/oemed-2023-108975

**Published:** 2024-06-26

**Authors:** Morgane Menant, Ilyes Benlala, Isabelle Thaon, Pascal Andujar, Benoist Julia, Patrick Brochard, Christos Chouaid, Bénédicte Clin, Antoine Gislard, Celine Gramond, Christophe Paris, Jean-Claude Pairon, Fleur Delva

**Affiliations:** 1 Bordeaux Population Health EPICENE, Bordeaux, France; 2 Faculté de Médecine, Université de Bordeaux, Talence, France; 3 Service d'Imagerie Thoracique et Cardiovasculaire, CHU Bordeaux, Pessac, France; 4 IINSPIIRE, Université de Lorraine, INSERM, Nancy, France; 5 Centre de Consultations de Pathologie professionnelle, CHU de Nancy, Nancy, France; 6 GEIC20 Team, INSERM U955, Créteil, France; 7 Service de Pathologies Professionnelles et de l’Environnement, Centre Hospitalier Intercommunal Créteil, Institut Santé-Travail Paris-Est, Créteil, France; 8 Institut Santé-Travail Paris-Est, Créteil, France; 9 IIMTPIF, Créteil, France; 10 Service Santé Travail Environnement, CHU de Bordeaux, Bordeaux, France; 11 Service de Pneumologie, CHI Créteil 94000 Créteil, Créteil, France; 12 Service de Santé au Travail et Pathologie Professionnelle, Caen, France; 13 INSERM U1086, Caen, France; 14 Faculté de Médecine, Normandie Université, UNIROUEN, UNICAEN, ABTE, Rouen, France; 15 Centre de consultations de pathologie professionnelle, Rouen cedex, France; 16 Service de Santé au Travail et Pathologie Professionnelle, Rennes, France; 17 INSERM U1085, Rennes, France

**Keywords:** Asbestos, Epidemiology, Occupational Health

## Abstract

**Objective:**

The aim of this study was to confirm the relationship between several parameters of exposure to asbestos and pleural plaques (PP) using data from a large cohort of retired workers occupationally exposed to asbestos in France.

**Method:**

A large screening programme, including high-resolution CT (HRCT) examinations at inclusion and two other HRCT campaigns, was organised from 2003 to 2016 in four regions of France for voluntary, formerly asbestos-exposed workers. Exposure to asbestos has been evaluated by industrial hygienists based on the complete work history. The time since first exposure, the time since last exposure, Cumulative Exposure Index and maximum level of exposure to asbestos, were used in logistic regression using fractional polynomials to model the relationship with PP.

**Results:**

The study included 5392 subjects with at least one HRCT available. There was a significant non-linear effect of time since first exposure, time since last exposure and Cumulative Exposure Index to asbestos on the presence of PP. The risk of PP increased with increasing Cumulative Exposure Index to asbestos adjusted for time since first exposure, age and smoking status. Models also show that PP odds rise with increasing time since first exposure adjusted for cumulative index exposure, age and smoking status. PP odds decrease when time since last exposure increases.

**Conclusion:**

The study provides new data on the link between asbestos exposure and the presence of PP using fractional polynomials with non-linear relationships for time exposure parameters and asbestos exposure parameters.

**WHAT IS ALREADY KNOWN ON THIS TOPIC**

- Asbestos exposure is known to be associated with pleural plaques (PP).

**WHAT THIS STUDY ADDS**

- There are non-linear relationships between time since first exposure and time since last exposure and the presence of PP.
- Definition of populations for screening of lung cancer is largely debated at present time (level of smoke, age…) and parameters of asbestos exposure and existence of pleural plaques might also be considered in the definition of eligible subjects.

**HOW THIS STUDY MIGHT AFFECT RESEARCH, PRACTICE OR POLICY**

- It appears important to carefully characterise the occurrence of PP to better define appropriate surveillance and screening programmes for these workers.

## Introduction

Asbestos is a natural mineral with a fibrous texture that was mined and industrially used for many decades in the 20th century.^1^ Exposure to asbestos can lead to the development of respiratory diseases. Most often, it causes benign diseases such as asbestosis or pleural plaques (PP), although malignant diseases can also occur (mesothelioma and lung, larynx and ovarian cancers).^1^ Although its use has been banned in many countries since the 1990s, the WHO estimates that 125 million workers were still exposed to it worldwide in 2018.^2^

PP are circumscribed areas of fibrous thickening, typically of the parietal pleura and diaphragmatic pleura. They are the most common disease after asbestos exposure and are considered a marker of asbestos exposure.^3^ Although benign, consideration of PP may be important since an association has been reported between the presence of PP and pleural mesothelioma adjusted to asbestos exposure.^4^ In addition, studies on a French cohort of asbestos-exposed subjects (the Asbestos Diseases COhort (ARDCO) have shown an increased risk of lung cancer in the presence of PP, confirmed by high-resolution CT (HRCT) adjusted to asbestos exposure.^5 6^ However, this association remains controversial.^7^

Numerous studies based on chest X-rays (CXR) have shown a positive association between the presence of PP and time since first asbestos exposure (TSFE),^8 9^ intensity of exposure^10–12^ or cumulative exposure to asbestos.^13 14^ However, CXR-based imaging does not easily identify non-calcified PP, which may influence previous results from CXR-based studies. Thoracic HRCT is considered to be the most specific and sensitive tool for PP diagnosis.^15 16^ There are fewer HRCT-based studies investigating the same associations, with inconsistencies between some of these studies. Eisenhawer *et al* 17 reported a significant association between the duration of exposure to asbestos and the presence of PP, while Mastrangelo *et al* did not.^18^ Similarly, in 2012, Ameille *et al* reported a non-significant effect of TSFE on the presence of PP,^19^ while other studies reported the opposite.^17 18^ Paris *et al* reported a significant association between TSFE and cumulative exposure to asbestos and the presence of PP.^20^

In 2009, Paris *et al* reported a significant non-linear effect of TSFE and Cumulative Exposure Index (CEI) to asbestos for the presence of PP in a French cohort study of asbestos-exposed subjects based on HRCT.^21^ The present study reported new data, based on follow-up of the same cohort with several HRCTs and improved characterisation of PP grounded in a double reading by expert radiologists.

The aim of the study was to confirm the relationship between asbestos exposure and PP using data from a large cohort of retired workers occupationally exposed to asbestos using HRCT data: ARDCO.

## Method

### Study design

A screening programme was conducted from October 2003 until December 2005, and included retired workers from four regions of France (Basse-Normandie, Haute-Normandie, Aquitaine and Rhône-Alpes), previously occupationally exposed to asbestos. The main purpose of this cohort was to improve medical surveillance of workers formerly exposed to asbestos. The ARDCO^21^ were recruited in four regions of France through the media (unsolicited applications), mail invitations sent to beneficiaries of the asbestos workers’ allowance, to beneficiaries of post-professional follow-up not renewed for more than 2 years, to inactive workers over 55 selected according to their occupational code, to early retirees according to their declared occupational category and by healthcare professionals. Volunteers were included if their exposure to asbestos was confirmed by an industrial hygienist according to information from their complete occupational calendar. At inclusion, subjects were offered a physical examination, HRCT and pulmonary function tests. Two additional HRCT campaigns were conducted in 2010 and 2016. Thus, all subjects included in the study had at least one available HRCT stocked on CD-ROM and no missing value on asbestos exposure variables of interest and smoking status.

### Data collection

A self-administered standardised questionnaire was used to collect socio-demographic characteristics (age, gender, sector of activity…), smoking status, and complete work history with title and duration for each job occupation mentioned.

### HRCT scanning

For each campaign, all HRCTs were read by two radiologists with expertise in thoracic imaging, and by a third radiologist in the event of disagreement. The radiologists did not know the subject’s cumulative asbestos exposure and were asked to complete a standardised form, including the diagnosis of PP and interstitial anomalies.^22^ Circumscribed quadrangular pleural elevations, with sharp borders and tissue density, sometimes calcified, led to a positive diagnosis of PP. If a subject had several HRCTs available, the first one with PP was selected for the study. For a subject with no PP, the last available HRCT was selected.

### Exposure assessment procedures

The complete work history of each subject was retrospectively analysed by industrial hygienists to evaluate asbestos exposure. All the information provided by the occupational questionnaire, including all successive jobs during the working life and specific questions on particular tasks, the context of the activities performed or contact with several asbestos-containing materials, was considered. The intensity of exposure was defined for each job and classified into four categories associated with a coefficient: low (0.01), low intermediate (0.1), high intermediate (1) and high (10). The maximum level of exposure for an individual corresponds to the maximum level found among all the individual’s jobs. A CEI to asbestos was then calculated for each subject over his/her working life, as the sum of exposures of each job (coefficient level × duration in years), expressed in exposure units × years since we had no metrology data. TSFE was defined as the time between the first year of exposure to asbestos and the date of the HRCT. Time since last exposure (TSLE) was defined as the time between the last year of exposure to asbestos and the date of the HRCT.

### Statistical analysis

The relationship between the parameters of occupational exposure to asbestos and the presence of PP was estimated using logistic regression models adjusted for age at HRCT and smoking status (never smokers, ex-smokers—having stopped for at least 1 year and current smokers). In model I, the parameters included for occupational asbestos exposure were CEI and TSFE. In model II, the parameters were maximum level exposure and TSFE. Models III and IV were respectively the same as models I and II, to which the TSLE variable was added.

For the association between TSFE, TSLE, age and CEI, and the presence of PP, the null hypothesis of linearity against alternative regression functions was tested and the best fitting model was selected. To this end, we used fractional polynomial regression models. This technique allows to model the effect of a continuous variable (TSFE, TSLE, age and CEI) on the result (presence of PP) by a non-linear fractional polynomial function if necessary.^23^

In all the models, 10 years was used as the reference value for the TSFE, 0 years was used as the reference for the TSLE and 25 unit-years was the reference value for the CEI. These reference values correspond to an approximate value of the minimum observed in our study population.

Statistical analyses were conducted using R software V.3.6.1.

## Results

### Cohort selection

**Figure 1:**
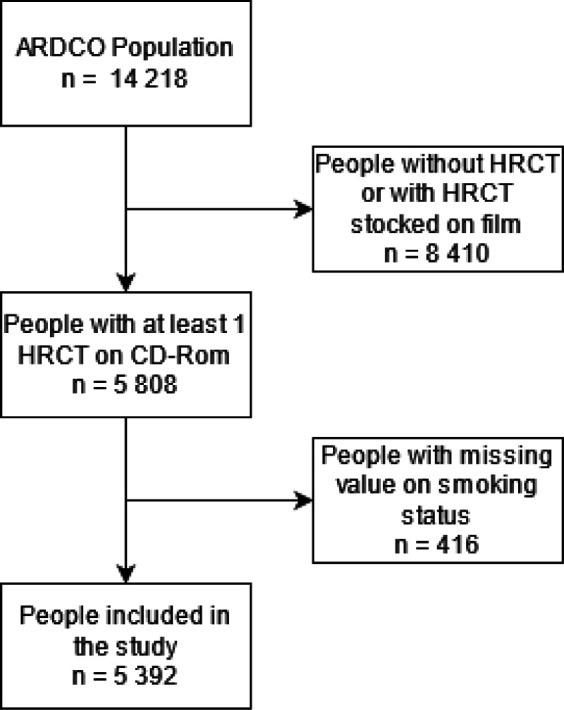
Study flow chart. ARDCO, Asbestos Diseases Cohort; HRCT, high-resolution CT.

Of the 14 218 subjects included in the ARDCO cohort, 5808 subjects had at least one CT stored on CD-ROM. Subjects with incomplete data on smoking status (n=416) were excluded from the analysis. Thus, 5392 subjects were included in the study (figure 1).

### Descriptive analysis

The characteristics of the study subjects are presented in table 1. The mean age at the HRCT scan was 66.9 years. The majority of the subjects (95.7%) were men and most of them were ex-smokers (64.2%). Regarding asbestos exposure, the majority of subjects (43.0%) were estimated to have a high intermediate maximum exposure level. Subjects with PP had a mean CEI to asbestos twice as high as those without (104.9 vs 55.2 units-year). Moreover, 44.8% of the subjects with PP had a ‘high’ maximum exposure level, while only 22.8% of the subjects without PP had a ‘high’ maximum exposure level to asbestos. The average time since first exposure was 47.4±8.7 years.

**Table 1:** Study population (N=5392)

Characteristics	All subjects (n=5392)	PP+ (n=1348)	PP− (n=4044)
Age at HRCT (years), mean±SD	66.9±7.2	67.2±6.6	66.9±7.4
Gender			
Men	5160 (95.7%)	1320 (97.9%)	3840 (95.0%)
Women	232 (4.3%)	28 (2.1%)	204 (5.0%)
Maximal exposure level to asbestos			
Low	232 (4.3%)	22 (1.6%)	210 (5.2%)
Low intermediate	1318 (24.4%)	193 (14.3%)	1125 (27.8%)
High intermediate	2316 (43.0%)	529 (39.2%)	1787 (44.2%)
High	1526 (28.3%)	604 (44.8%)	922 (22.8%)
CEI to asbestos (units-year), mean±SD	67.7±102.8	104.9±122.9	55.2±91.9
Time since first exposure (years), mean±SD, min — max	47.4±8.7 8 — 74	48.4±7.6 9 — 74	47.1±9.1 8 — 74
Time since last exposure (years), mean±SD, min — max	15.0±9.9 0 — 62	14.3±9.8 0 — 54	15.3±9.9 0 — 62
Smoking status			
Smoker	412 (7.6%)	99 (7.3%)	323 (7.7%)
Ex-smoker	3463 (64.2%)	951 (70.5%)	2512 (62.1%)
Non-smoker	1517 (28.1%)	298 (22.1%)	1219 (30.1%)

CEI, Cumulative Exposure Index; HRCT, high-resolution CT; PP, pleural plaques.

The prevalence of PP among the 5392 subjects was 25% (n=1348), reaching 39.6% among subjects with ‘high’ maximum exposure level.

Interstitial abnormalities (minor abnormalities, abnormalities incompatible with common interstitial lung disease, possible or certain common interstitial lung disease or asbestosis) were present in 833 subjects (15.4%) and, more specifically, in 540 subjects without PP (13.3%) and 293 subjects with PP (21.7%). If we consider only possible or certain common interstitial lung disease or asbestosis, they were present in 1.7% of subjects, and more precisely in 1.2% of subjects without PP and in 3.3% of subjects with PP.

### Multivariate analyses

The results of the multivariate analyses show that all of the asbestos exposure parameters studied were associated with the presence of PP (table 2).

**Table 2:** Association between parameters of exposure to asbestos and pleural plaques (N=5392)

	Univariate model	Multivariate models			
		Model I	Model II	Model III	Model IV
	OR (95% CI)	OR (95% CI)	O (95% CI)	OR (95% CI)	OR (95% CI)
Time since last exposure (10 years)	**0.91 (0.85 to 0.97**)				
Time since last exposure (years)					
10 vs 0				**0.69 (0.58 to 0.82**)	**0.65 (0.54 to 0.77**)
20 vs 0				**0.58 (0.44 to 0.76**)	**0.50 (0.38 to 0.65**)
30 vs 0				**0.60 (0.44 to 0.82**)	**0.45 (0.33 to 0.62**)
40 vs 0				0.76 (0.55 to 1.05)	**0.49 (0.36 to 0.68**)
50 vs 0				1.19 (0.79 to 1.79)	**0.64 (0.42 to 0.96**)
Time since first exposure (years)					
20 vs 10	**2.69 (2.08 to 3.49**)	**1.91 (1.38 to 2.66**)	**1.95 (1.41 to 2.69**)	**1.98 (1.43 to 2.75**)	**2.03 (1.47 to 2.81**)
30 vs 10	**7.17 (4.32 to 11.90**)	**3.69 (1.95 to 7.01**)	**3.87 (2.05 to 7.27**)	**3.94 (2.08 to 7.48**)	**4.19 (2.23 to 7.88**)
40 vs 10	**15.09 (7.61 to 29.91**)	**6.21 (2.62 to 14.70**)	**6.73 (2.87 to 15.75**)	**6.73 (2.84 to 15.96**)	**7.48 (3.19 to 17.52**)
50 vs 10	**21.48 (10.13 to 45.55**)	**8.22 (3.18 to 21.26**)	**9.29 (3.63 to 23.70**)	**8.88 (3.43 to 23.03**)	**10.40 (4.06 to 26.59**)
60 vs 10	**18.33 (9.21 to 36.45**)	**7.93 (3.24 to 19.40**)	**9.38 (3.87 to 22.71**)	**8.35 (3.40 to 20.49**)	**10.35 (4.27 to 25.11**)
70 vs 10	**7.42 (4.32 to 12.74**)	**5.24 (2.40 to 11.44**)	**6.53 (3.00 to 14.19**)	**5.22 (2.39 to 11.45**)	**6.90 (3.17 to 15.03**)
CEI to asbestos (units-year)					
100 vs 25	**1.69 (1.59 to 1.79**)	**1.81 (1.67 to 1.96**)		**1.84 (1.69 to 1.99**)	
200 vs 25	**2.17 (1.96 to 2.41**)	**2.37 (2.13 to 2.64**)		**2.41 (2.16 to 2.69**)	
300 vs 25	**2.45 (2.09 to 2.88**)	**2.55 (2.17 to 2.99**)		**2.57 (2.19 to 3.02**)	
400 vs 25	**2.60 (2.08 to 3.26**)	**2.47 (1.92 to 3.18**)		**2.46 (1.91 to 3.17**)	
Maximal exposure level					
Low (n=232)	**1**		**1**		**1**
Low intermediate (n=1318)	**1.63 (1.03 to 2.61**)		1.53 (0.96 to 2.45)		1.47 (0.92 to 2.35)
High intermediate (n=2316)	**2.82 (1.80 to 4.43**)		**2.58 (1.64 to 4.08**)		**2.49 (1.58 to 3.93**)
High (n=1526)	**6.25 (3.98 to 9.82**)		**6.03 (3.82 to 9.52**)		**5.93 (3.75 to 9.37**)

Model I: Association between Cumulative Exposure Index of asbestos, time since first exposure and the presence of pleural plaques, adjusted for age and smoking status. Model II: Association between time since first exposure to asbestos, maximal level of exposure and the presence of pleural plaques, adjusted for age and smoking status. Model III: Association between Cumulative Exposure Index of asbestos, time since first exposure, time since last exposure to asbestos and the presence of pleural plaques adjusted for age and smoking status. Model IV: Association between time since first exposure to asbestos, time since last exposure to asbestos, maximal level of exposure and the presence of pleural plaques, adjusted for age and smoking status.

Bold values: p<0.05.

CEI, Cumulative Exposure Index.

A non-linear effect of age, TSFE, TSLE and CEI on the presence of PP was observed and therefore modelised using fractional polynomials.

Model I shows that TSFE and CEI are significantly and independently associated with the prevalence of PP, adjusted for age and smoking status.

**Figure 2:**
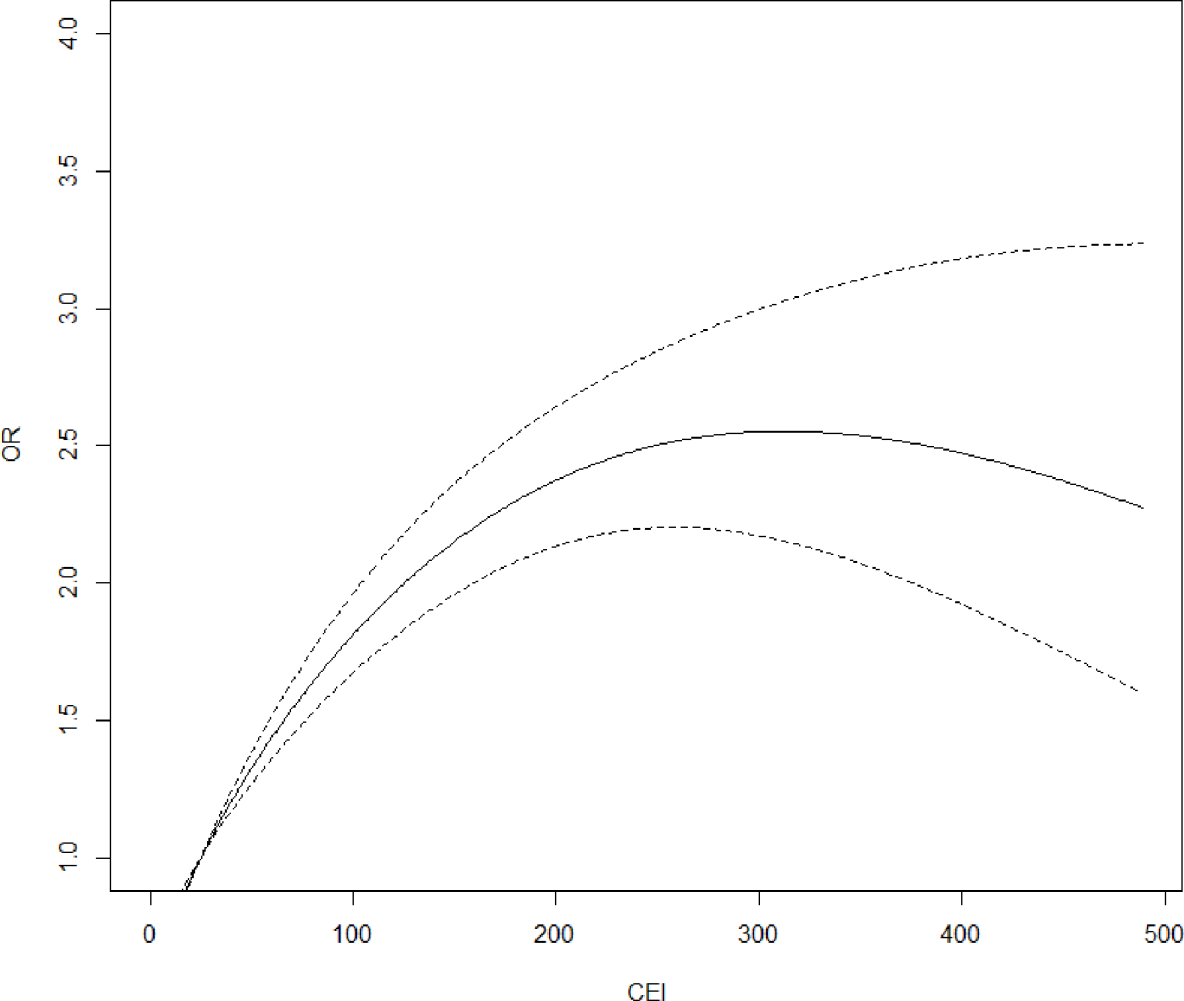
Pleural plaques ORs as a function of Cumulative Exposure Index (CEI), reference=25 units-years. Model I: logistic regression model, effect of CEI to asbestos, and time since first exposure to asbestos on presence of pleural plaques, adjusted for age and smoking status, n=5392.

Specifically, Model I shows that PP odds rise with increasing CEI and reach a maximum for values of around 300 unit-years: OR_300 VS 25 units-years_=2.55 (95% CI 2.17 to 2.99). For values above 300 unit-years, the ORs for PP appear to remain roughly constant (figure 2).

**Figure 3:**
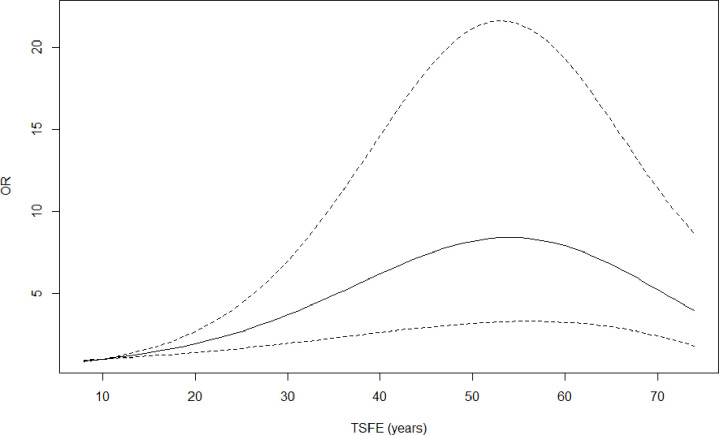
Pleural plaques ORs as a function of time since first exposure (TSFE), reference=10 years. Model I, logistic regression model, effect of cumulative index exposure to asbestos, and time since first exposure to asbestos on presence of pleural plaques, adjusted for age and smoking status, n=5392.

Model I also shows that PP odds increase with increasing TSFE up to a TSFE value of about 50 years: OR_50 VS 10 years_=8.22 (95% CI 3.18 to 21.26) (figure 3).

Model II shows that TSFE and the maximum levels of asbestos exposure ‘high intermediate’ and ‘high’ (reference ‘low’) are also significantly and independently associated with the prevalence of PP, adjusted for age and smoking status. The effect of TSFE on the presence of PP is similar to model I (online supplemental figure S1).

10.1136/oemed-2023-108975.supp1 Supplementary data

Models III and IV show that PP odds decrease when TSLE increases (online supplemental figures S2 and S3) for TSLE values up to 30 years (reference 0), adjusted to CEI, TSFE, age and smoking status for model III and adjusted to TSFE, maximum exposure level, age, and smoking status for model IV.

## Discussion

The main result of this study concerns the non-linear effect of asbestos exposure determinants associated with the presence of PP for both time (TSFE and TSLE) and dose (CEI or maximal exposure level) parameters. The study confirms the previous findings for this population,^21^ with a finer categorisation of the exposure, a longer follow-up of the subjects with an additional and double expert reading of HRCT, and a more precise characterisation of the effects. It suggests that TSLE is associated with a decrease in the likelihood of having PP.

### Dose and time effects

The study showed a non-linear effect of CEI on the PP odds. The risk of PP increases with CEI and reaches a maximum before stabilising at around 300 units-years. Other HRCT-based studies have shown a non-linear effect of CEI on PP odds modelling, using classes of CEI.

Some studies, like ours, have a study population with heterogeneous exposure to asbestos, involving several sectors of activity. Mastrangelo *et al* showed a significant effect of CEI for dose classes above 159 fibres/mL-year compared with 43 fibres/mL-year adjusted for age and smoking status : OR_43–159 fibres/mL-year_ = 1.99 (95% CI 1.20 to 3.28)).^18^ Murray *et al* showed that for every unit increase of asbestos exposure in fibres/mL-year, the plaque score increased by 0.074 (95% CI 0.048 to 0.099).^24^ The plaque score considered the circumference of the plaques in relation to the rib cage and their thickness, and was 0 in subjects without PP.

Other studies are based on cohort data with more homogeneous exposure. Rapisarda *et al* reported that the prevalence ratio of PP increased with increasing CEI in fluoro-edenite exposed workers.^25^ Eisenhawer *et al* 17 studied workers in electrical energy and reported a significant effect of CEI on PP odds for exposure above 25 fibres/year compared with one fibre/year, adjusted for smoking and age. However, in Eisenhawer *et al*’s study, this effect was not found in the analyses including temporal variables such as TSFE or exposure duration. Modelling a non-linear association using classes rather than functions such as fractional polynomials may explain the model’s difficulty in showing the effects of dose and of time.^26^ Lockey *et al* who studied workers exposed to Libby vermiculite also showed that cumulative exposure to asbestos fibres was significantly associated with pleural changes, including diffuse and localised pleural thickening for exposure above <0.15 fibre-year/cm^3^.^27^ Ameille *et al*, who studied transport workers with low exposure to asbestos, did not find a statistical association between CEI to asbestos and the presence of PP (p=0.26).^28^

Barbieri *et al* published autopsy analyses in 2019 that support the links demonstrated in this and other studies between cumulative asbestos exposure and the presence of PP.^29^

In addition, our study showed a non-linear effect of TSFE on the PP odds. PP odds increase with TSFE to reach a maximum of around 50 years. Graphically, we can thus observe a stabilisation. Interpretation of the apparent decrease in the effect of TSFE on the presence of PP for values beyond 60 years should be approached with caution given the number of subjects and thus the power of the model for these values. Other HRCT-based studies have investigated the effect of TSFE on the prevalence of PP. Eisenhawer *et al* showed a linear and significant effect of TSFE based on data from a cohort of electric power workers : OR=1.61 (95% CI 1.07 to 2.41).^17^ In a study of transport workers with low exposure to asbestos, Ameille *et al* did not find this association (p=0.56).^28^

Among studies with heterogeneous asbestos exposure study populations and data from HRCT, Mastrangelo *et al* found a non-linear effect of TSFE on the presence of PP modelled by classes: OR_27–30 VS ≤26 years_ = 3.64 (95% CI 2.17 to 6.11).^18^

With regard to TSLE, the effect on the presence of PP presented in our study is adjusted for cumulative asbestos exposure or maximum exposure level, and is therefore significant irrespective of asbestos exposure dose. However, it is possible that a selective survival bias may have occurred. Indeed, compared with less-exposed individuals, those most exposed to asbestos are more likely to develop PP, but also have a higher risk of developing and dying from other diseases such as lung cancer or mesothelioma, and therefore of not being able to be followed up as long as those with less severe damage. It is therefore possible that, in this type of population, a longer TSLE is observed in subjects without PP than in those with PP, partly due to this survival bias. In such hypothesis, the conclusion about the association of TSLE and PP could not be applied to subjects highly exposed to asbestos.

### Strengths and limitations

The present study modelled the non-linear effects of quantitative variables on the presence of PP using fractional polynomials, which allow for a more accurate account of their effect than classes.

Moreover, asbestos exposure measurement was assessed by industrial hygienists using the occupational calendar, which provided an expertise covering the subjects’ entire career. This collection method may involve memory bias. In the case of our study, the professional calendar data were collected before the CT examinations, we consider that the exposure history was not influenced by the diagnosis of PP.

The PP diagnosis was made by HRCT, with a double or triple reading in the event of disagreement between experts, helping to limit classification errors.

The prevalence of PP in all subjects in our study was 25%. Since exposure to asbestos of subjects in this study covered a wide range of industrial activities, the level of CEI was significantly variable. However, the prevalence of PP reached 39.6% in subjects with a ‘high’ maximum exposure level.

The cohort included subjects on a voluntary basis, so we can suspect that subjects with the highest exposure to asbestos were already being monitored by a physician or pneumologist with possible asbestos-related disease, as they were more likely to develop symptoms and so did not respond to inclusion. The results of our study may therefore not be transposable to a population highly exposed to asbestos. Finally, as HRCT was performed at a given point in time, the TSFE may have been overestimated in the event that the PP appeared before the diagnostic examination was performed. Thus, it is possible that the times after first exposure to asbestos presented in this study were slightly overestimated in a fraction of the subjects.

## Conclusion

Based on a large French CT-based screening programme in asbestos-exposed subjects, the study provided new data on the link between asbestos temporal parameters and the presence of PP. Indeed, using fractional polynomials, it described non-linear relationships between TSFE and TSLE and the presence of PP. Notably, TSLE appears to show that cessation of exposure is associated with a decrease in the likelihood of developing PP. Time since cessation of exposure to asbestos could be considered in the follow-up of populations exposed to asbestos, in particular in designing the screening programme for lung cancer. Indeed, as a link is suspected between the presence of PP and excess risk of lung cancer in asbestos-exposed workers, it appears important to carefully characterise the occurrence of PP in order to better define appropriate surveillance and screening programmes for these workers.

## Data Availability

No data are available.
