# Supplementary material for: Relationships between asbestos exposure and pleural plaques: dose and time effects using fractional polynomials
Source: Occup Environ Med. 2024 Jun 26;81(6):313–9. doi: 10.1136/oemed-2023-108975 (PMC11287532; doi:10.1136/oemed-2023-108975)
Supplement: Supplementary data [file oemed-2023-108975supp001.pdf]

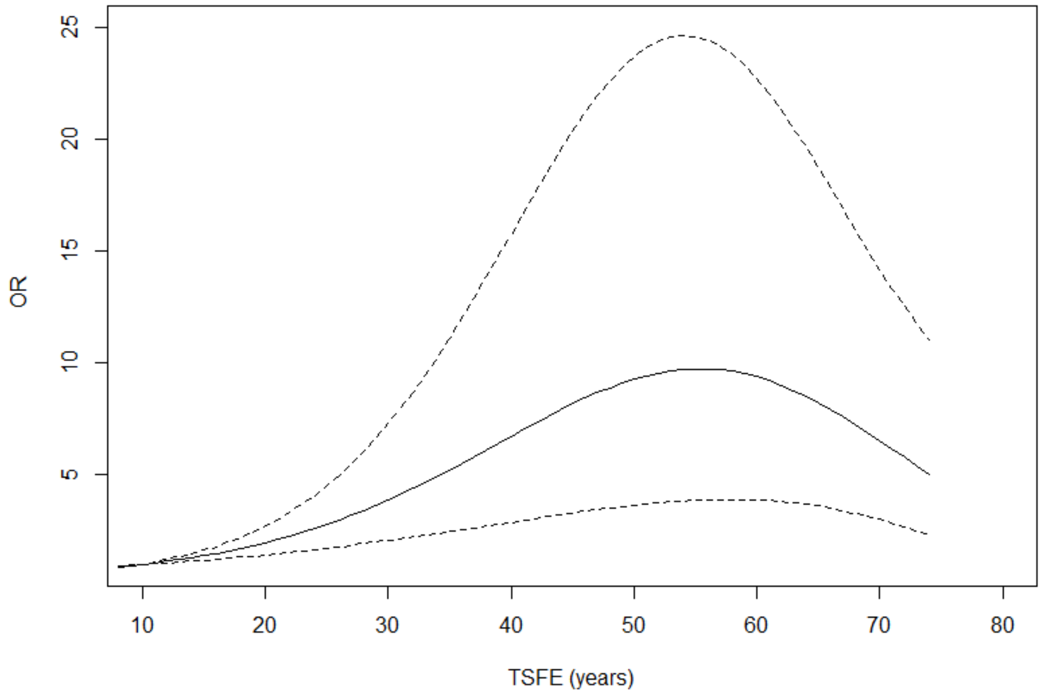

Supplementary material Figure S1 - Pleural plaques odds ratios as a function of time since first exposure (TSFE), reference = 10 years. Model II, logistic regression model, effect of maximal exposure level to asbestos, and time since first exposure to asbestos on presence of pleural plaques, adjusted for age and smoking status n=5,392.

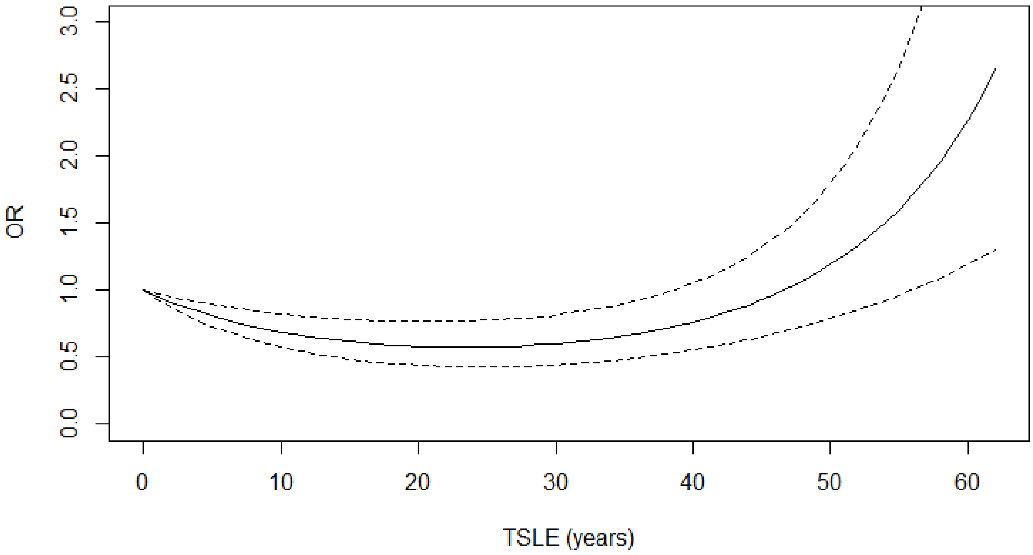

Supplementary material Figure S2 - Pleural plaques odds ratios as a function of time since last exposure (TSLE), reference = 0 year. Model III, logistic regression model, effect of cumulative index of exposure to asbestos, time since first exposure to asbestos, and time since last exposure to asbestos on presence of pleural plaques, adjusted for age and smoking status, n=5,392.

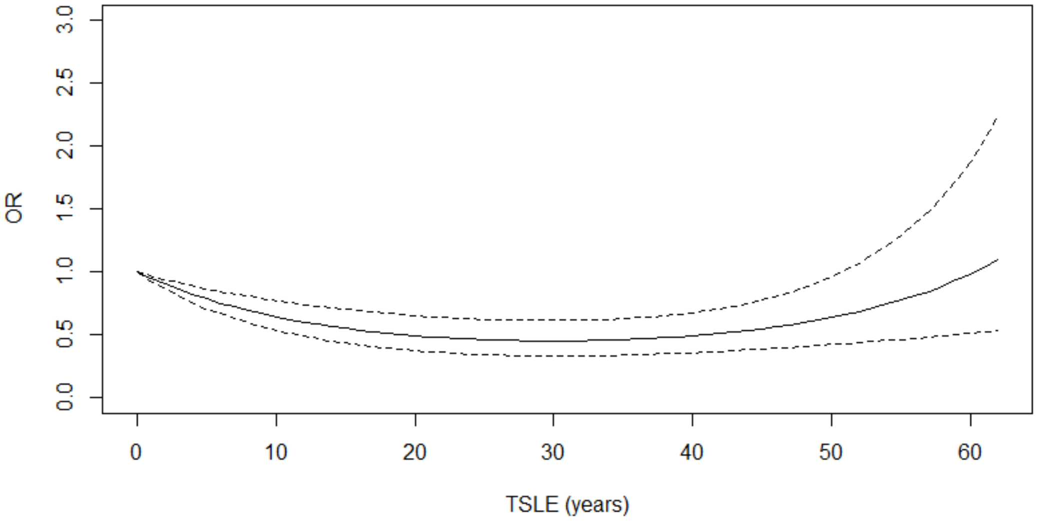

Supplementary material Figure S3 - Pleural plaques odds ratios as a function of time since last exposure (TSLE), reference = 0 year. Model IV, logistic regression model, effect of maximum exposure level to asbestos, time since first exposure to asbestos, and time since last exposure to asbestos on presence of pleural plaques, adjusted for age and smoking status, n=5,392.
